# Annexin A2 Egress during Calcium-Regulated Exocytosis in Neuroendocrine Cells

**DOI:** 10.3390/cells9092059

**Published:** 2020-09-09

**Authors:** Marion Gabel, Cathy Royer, Tamou Thahouly, Valérie Calco, Stéphane Gasman, Marie-France Bader, Nicolas Vitale, Sylvette Chasserot-Golaz

**Affiliations:** 1 Centre National de la Recherche Scientifique, Université de Strasbourg, Institut des Neurosciences Cellulaires et Intégratives, F-67000 Strasbourg, France; m.gabel@wanadoo.fr (M.G.); tam@inci-cnrs.unistra.fr (T.T.); calco@unistra.fr (V.C.); gasman@inci-cnrs.unistra.fr (S.G.); badermf@inci-cnrs.unistra.fr (M.-F.B.); vitalen@inci-cnrs.unistra.fr (N.V.); 2 Plateforme Imagerie In Vitro, Neuropôle, Université de Strasbourg, F-67000 Strasbourg, France; cathy.royer@unistra.fr

**Keywords:** annexinA2 egress, exocytosis, chromaffin cells

## Abstract

Annexin A2 (AnxA2) is a calcium- and lipid-binding protein involved in neuroendocrine secretion where it participates in the formation and/or stabilization of lipid micro-domains required for structural and spatial organization of the exocytotic machinery. We have recently described that phosphorylation of AnxA2 on Tyr^23^ is critical for exocytosis. Considering that Tyr^23^ phosphorylation is known to promote AnxA2 externalization to the outer face of the plasma membrane in different cell types, we examined whether this phenomenon occurred in neurosecretory chromaffin cells. Using immunolabeling and biochemical approaches, we observed that nicotine stimulation triggered the egress of AnxA2 to the external leaflets of the plasma membrane in the vicinity of exocytotic sites. AnxA2 was found co-localized with tissue plasminogen activator, previously described on the surface of chromaffin cells following secretory granule release. We propose that AnxA2 might be a cell surface tissue plasminogen activator receptor for chromaffin cells, thus playing a role in autocrine or paracrine regulation of exocytosis.

## 1. Introduction

Molecules such as neurotransmitters and hormones are secreted by calcium-regulated exocytosis [1,2]. In neuroendocrine cells, exocytosis implies the recruitment and subsequent fusion of secretory granules at specific sites of the plasma membrane. The scaffolding protein annexin A2 (AnxA2) is a promoter of these sites of exocytosis in cells activated for secretion. AnxA2 binds two major actors of exocytosis, actin and phospholipids. In chromaffin cells, electron tomography has revealed that actin filaments bundled by AnxA2 contribute to the formation of lipid micro-domains at the plasma membrane required for the spatial and functional organization of the exocytotic machinery [3,4,5]. More recently, we have found that AnxA2 needs to be phosphorylated on Tyr^23^ to stabilize the lipid platform determining the exocytotic site, and then dephosphorylated to bundle actin filaments for stably anchoring granules, implying that the phosphorylation cycle of AnxA2 on Tyr^23^ is critical for neuroendocrine secretion [6].

In some cell types, one of the consequences of the AnxA2 phosphorylation is the egress of AnxA2, i.e., its passage through the plasma membrane. For instance, AnxA2 appeared on the cell surface of endothelial cells following a thermal shock [7], calcium-stimulated fibroblasts [8] or depolarized cortical neurons [9]. In neurons, cell surface AnxA2 was found to interact with tissue plasminogen activator (t-PA), which is involved in synaptic plasticity and memorization [10] as well as in neuronal death via plasmin proteolysis activity [11]. In chromaffin cells, one study reported that AnxA2 is “secreted” unconventionally in the extracellular medium following nicotine stimulation [12]. This release of AnxA2 was correlated with catecholamine secretion and found to be independent of cell death.

Having recently shown that the AnxA2 is phosphorylated on Tyr^23^ during exocytosis [6], we examined whether Tyr^23^ phosphorylation of AnxA2 could lead to its externalization in chromaffin cells. Using immunolabeling and biochemical approaches, we show here that nicotine stimulation triggers the egress of AnxA2 to the external leaflets of the plasma membrane and identify tissue plasminogen activator (t-PA) as a potential partner at the chromaffin cell surface.

## 2. Materials and Methods

### 2.1. Antibodies and Reagents

Rabbit polyclonal antibodies directed against AnxA2 purified from bovine aorta were a generous gift from J.C. Cavadore (Inserm U-249) [13]. Monoclonal antibodies anti-AnxA2 and anti-pTyr^23^-AnxA2 (85.Tyr24) were purchased respectively from BD Transduction Laboratories (Pont de Claix, France) and Santa Cruz Biotechnology Inc. (Dallas, TX, USA). Mouse monoclonal antibodies directed against dopamine ß-hydroxylase (EC.1.14.17.1: DBH), used to specifically label secretory granules in chromaffin cells [14], were purchased from Sigma-Aldrich (St. Louis, MO, USA) and the rabbit polyclonal anti-chromogranin A (CgA) from Abcam (Amsterdam, Netherlands). Rabbit polyclonal anti-mouse tissue plasminogen activator (t-PA) antibodies were from Molecular Innovations (Novi, MI, USA). Mouse monoclonal antibodies against S100A10 were from Transduction Laboratories (Lexington, KY, USA). Sheep polyclonal antibodies against bovine phenyl ethanolamine *N*-transferase (PNMT) were purchased from Chemicon International Inc., (Temecula, CA, USA) and TRITC- or Atto-647 *N*-phalloidin from Sigma-Aldrich (St. Louis). Rabbit polyclonal anti-CD63 antibodies were from Santa Cruz Biotechnology Inc (Dallas, TX, USA)**.** Secondary antibodies coupled to Alexa Fluor^®^ conjugates (488, 561 or 647) or gold particles were from Molecular Probes (Invitrogen, Cergy Pontoise, France) and Aurion (Wageningen, Netherlands) respectively.

The construct allowing for the simultaneous expression of the catalytic subunit of the tetanus toxin (TTx) and the GFP was generated by subcloning both the eGFP and the TTx (residues 1 to 457, a generous gift from Thomas Binz [15]) into a bidirectional expression vector. The eGFP was PCR amplified as a BglII/PstI fragment using 5′-TATAGATCTCGCCACCATGGTGAGCAAGGGCGA-3′ and 5′-CGCCTGCAGTTACTTGTACAG CTCGTCCATGC-3′ primers and cloned into the MCS2 of the pBI-CMV1 vector (TAKARA Bio USA, Mountain View, CA, USA). Then, the TTx was PCR amplified as an MluI/NotI fragment using 5′-TATACGCGTGCCACCATGCCGATCACCATCAAC-3′ and 5′-TATGCGGCCGCTTAAGCGGT ACGGTTGTACAG-3′ primers and cloned into the MCS1 of the pBI-GFP vector. The botulinum C toxin (Bot C Tx) plasmid expressing the light chain of the toxin [16] was co-transfected with pmaxGFP to identify cells expressing the toxin.

### 2.2. Chromaffin Cell Culture and Transfection

Chromaffin cells were isolated from fresh bovine adrenal glands by perfusion with collagenase A, purified on self-generating Percoll gradients and maintained in culture as previously described [17]. To induce exocytosis, chromaffin cells were washed twice with Locke’s solution (140 mM NaCl, 4.7 mM KCl, 2.5 mM CaCl_2_, 1.2 mM KH_2_PO_4_, 1.2 mM MgSO_4_, 11 mM glucose, 0.56 mM ascorbic acid, 0.01 mM ethylene diamine tetraacetic acid (EDTA) and 15 mM Hepes, pH 7.5), and then stimulated either with Locke’s solution containing 20 µM nicotine or high K^+^ solution (86,9 mM NaCl, 59 mM KCl, 2.5 mM CaCl_2_, 1.2 mM KH_2_PO_4_, 1.2 mM MgSO_4_, 11 mM glucose, 0.56 mM ascorbic acid, 0.01 mM EDTA and 15 mM Hepes, pH 7.2).

TTx-GFP was transfected into chromaffin cells (5 × 10^6^ cells) by electroporation (Amaxa Nucleofactor systems, Lonza, Levallois, France) according to the manufacturer’s instructions. Electroporated cells were immediately recovered in warm culture medium and plated onto fibronectin-coated glass coverslips. Experiments were performed 48 h after transfection.

### 2.3. Cell Stimulation and Extracellular AnxA2 and t-PA Measurements

Two days after plating in 5 cm Petri dishes, 10 × 10^6^ chromaffin cells were washed for 5 min with Locke’s solution, then 5 min with Locke’s solution without Ca^2+^ (140 mM NaCl, 4.7 mM KCl, 1.2 mM KH_2_PO_4_, 1.2 mM MgSO_4_, 0.25 mM ethylene glycol-tetraacetic acid (EGTA), 1 mM glucose, 125 µM ascorbate and 15 mM HEPES, pH 7.5) to discard cellular debris. Cells were then stimulated for 5 min with 1 mL of either 20 µM nicotine in Locke’s solution or of high K^+^ solution. Similarly, control cells were treated with Locke’s solution. The media of stimulated cells were recovered (fraction “secreted material”) and the cells were further treated for 5 min with calcium-free Locke’s solution containing 20 mM EGTA (fraction “EGTA eluate”). Finally, cells were scraped into 500 µL lysis buffer (Cell Extraction Buffer, Novex^®^, Fisher Scientific, Illkirch-Graffenstaden, France) supplemented with protease inhibitor cocktail (P8340, Sigma-Aldrich, St. Louis). The lysates were sonicated and centrifuged and the supernatants were recovered. Secreted material and the EGTA eluate fractions were concentrated 10 times to obtain 50 µL (Spin-X UF, Corning, Wiesbaden, Germany) for AnxA2 and t-PA detection. Aliquots of supernatants were used for the lactate dehydrogenase (LDH) activity assay (QuantiChromTM LDH, D2DH-100, BioAssay Systems, Hayward, CA, USA). To isolate extracellular vesicles, the secreted material was centrifuged at 300× *g* for 10 min at 4 °C to discard cellular debris, then the supernatant was centrifuged at 100,000× *g* for 2 h at 4 °C. The pellet was suspended in 15 µL of lane marker reducing sample buffer (Thermo Scientific, Illkirch Graffenstaden, Franch). Then the samples were separated on a 4–12% SDS-PAGE gel (Novex, Thermo Scientific), blotted to nitrocellulose with the Trans-blot Turbo System (Biorad, Marnes-la Coquette, Franch) and revealed with Substrat chemiluminescent SuperSignal™ (West Femto, Thermo Scientific).

### 2.4. Immunofluorescence and Confocal Microscopy

For immunocytochemistry, chromaffin cells, grown on fibronectin-coated glass coverslips, were fixed and labeled as described previously [14]. The transient accessibility of DBH to the plasma membrane of chromaffin cells was tested by incubating cells for 5 min in Locke’s solution containing 20 µM nicotine and anti-DBH antibodies diluted to 1:100. F-actin was stained with TRITC-phalloidin (0.5 µg/mL) for 15 min in the dark at room temperature. Labeled cells were visualized using a Leica SP5II confocal microscope. Nonspecific fluorescence was assessed by incubating cells with the secondary fluorescent-conjugated antibodies. To compare the labeling of cells from different conditions within the same experiment, images were acquired at the equatorial plane of the nucleus with the same parameters of the lasers and photomultipliers. The amount of AnxA2 or t-PA labeling associated with the plasma membrane was measured with ICY software [18] and expressed as the average fluorescence intensity normalized to the labeling surface, and divided by the total area of each cell. This allowed a quantitative cell-to-cell comparison of the fluorescence detected in cells.

### 2.5. Plasma Membrane Sheet Preparation and Transmission Electron Microscopy Observation

Cytoplasmic face-up membrane sheets were prepared and processed as previously described [19]. Briefly, carbon-coated Formvar films on nickel electron grids were inverted onto unstimulated or nicotine-stimulated chromaffin cells incubated with antibodies. To prepare membrane sheets, pressure was applied to the grids for 20 s, then the grids were lifted so that the fragments of the upper cell surface adhered to the grid. These membrane portions were fixed in 2% paraformaldehyde for 10 min at 4 °C. After blocking in PBS with 1% BSA and 1% acetylated BSA, the immune labeling was performed and revealed with gold particle-conjugated secondary antibodies. These membrane portions were fixed in 2.5% glutaraldehyde in PBS, postfixed with 0.5% OsO4, dehydrated in a graded ethanol series, treated with hexamethyldisilazane (Sigma-Aldrich, St. Louis), air-dried and observed using a Hitachi 7500 transmission electron microscope.

### 2.6. Statistical Analysis

As specified in figure legends, groups of data are presented as mean (±SEM) or median and were analyzed using a Mann-Whitney test. Asterisks in each box and whisker plot indicate statistical significance.

## 3. Results

### 3.1. AnxA2 Crosses the Plasma Membrane in Stimulated Chromaffin Cells in a Ca^2+^-Dependent Manner and Accumulates on the Extracellular Membrane Leaflet

**Figure 1:**
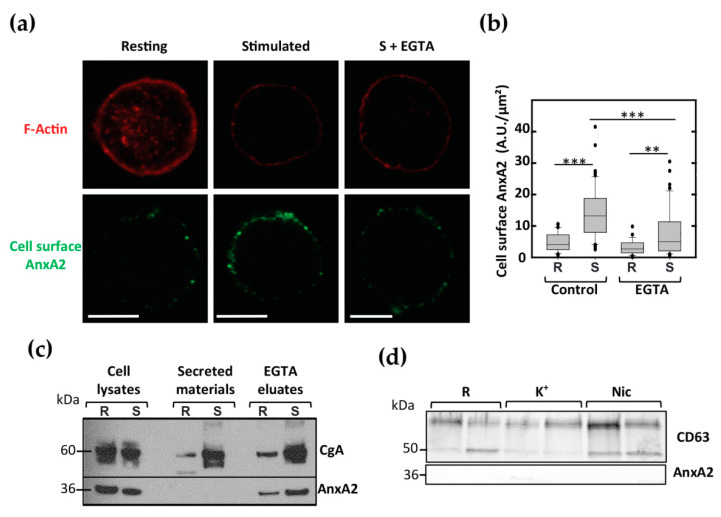
Annexin A2 (AnxA2) is present on the outer face of the plasma membrane of stimulated chromaffin cells. (**a**) AnxA2 labeling at the surface of chromaffin cells in the resting condition (Resting), stimulated with 20 µM nicotine without (Stimulated) or after washing with calcium-free Locke’s solution containing 20 mM EGTA (Stimulated + EGTA). Anti-AnxA2 antibodies were revealed with Alexa Fluor^®^488-conjugated anti-rabbit antibodies and F-actin with TRITC-phalloidin. Confocal images were recorded in the same optical section by a dual exposure procedure with the same parameters of lasers and photomultipliers. Scale bar: 10 µM. (**b**) Semi-quantitative analysis of cell surface AnxA2 labeling in chromaffin cells in resting condition (R) or stimulated with 20 µM nicotine (S) without (Control) or after EGTA wash (EGTA). AnxA2 labeling is expressed in arbitrary units. Statistical significance for medians was determined using a Mann-Whitney test. Dotted lines indicate the mean and asterisks statistical significance (*** = *p* < 0.001, ** = *p* < 0.01). Three experiments were done on independent cell cultures and pooled (*n* = 32 and 63 control cells, 28 and 63 EGTA-treated cells for resting and stimulated conditions, respectively). (**c**) Lysate, secreted material and EGTA eluate from chromaffin cells in the resting condition (R), or stimulated with 20 µM nicotine (S) were analyzed by western blot and revealed with anti-CgA and anti-AnxA2 antibodies. Data correspond to a typical experiment representative of three independent experiments. (**d**) The 100,000 g pellet of secreted materials from chromaffin cells in the resting condition (R), or stimulated with high K^+^ solution (K^+^) or 20 µM nicotine (Nic) were analyzed by western blot and revealed with anti-CD63 and anti-AnxA2 antibodies. Samples analyzed were obtained from two independent experiments.

Bovine chromaffin cells represent a good model to study regulated exocytosis [20]. They express various nicotinic receptors and accordingly exocytosis is triggered by nicotinic agonists [21,22]. To examine whether AnxA2 could be found on the cell surface of stimulated chromaffin cells, living chromaffin cells maintained under resting conditions or stimulated with nicotine were incubated in the presence of anti-AnxA2 antibodies to specifically label AnxA2 potentially present on the cell surface. Cells were then fixed and labeled with phalloidin-TRITC to reveal the actin cytoskeleton. Resting cells characterized by a typical F-actin ring at the cell periphery displayed only faint cell surface AnxA2 labeling (Figure 1a). In contrast, while F-actin labeling decreased in stimulated cells in line with the actin depolymerization occurring during exocytosis, the labeling of AnxA2 on the cell surface increased (Figure 1a). In some experiments, cells stimulated with nicotine in the presence of the AnxA2 antibodies were further washed with a calcium-free Locke’s solution containing 20 mM EGTA to collect proteins bound in a calcium-dependent manner to the cell surface (S + EGTA). Washing cells with EGTA led to a diminution of the cell surface AnxA2 labeling. Semi-quantitative analysis of the confocal images (Figure 1b) indicated that cell stimulation increased by approximately three times the amount of AnxA2 detected on the surface of chromaffin cells and confirmed the calcium sensitivity of AnxA2 binding to the plasma membrane.

Next, a biochemical approach was performed using fractions collected from nicotine-stimulated chromaffin cells. As illustrated in Figure 1c, AnxA2 was not found in the secreted material containing chromogranin A (CgA), but it could be detected in the calcium-free EGTA solution (EGTA eluates) used to wash cells after stimulation and in the cell lysates. In line with the absence of AnxA2 in secretory granules, these data suggest that AnxA2 is not secreted via the conventional exocytotic pathway but it is found bound in a calcium-dependent manner on the extracellular face of the plasma membrane following cell stimulation. Moreover, we did not detect AnxA2 in 100,000 g pellets of secreted materials (Figure 1d) containing the specific marker of extracellular vesicle CD63 [23]. Thus, AnxA2 did not seem to be associated with the extracellular vesicles released after cell stimulation.

### 3.2. Tyr ^23^ Phosphorylated AnxA2 Tetramer Binds the External Face of the Plasma Membrane

**Figure 2:**
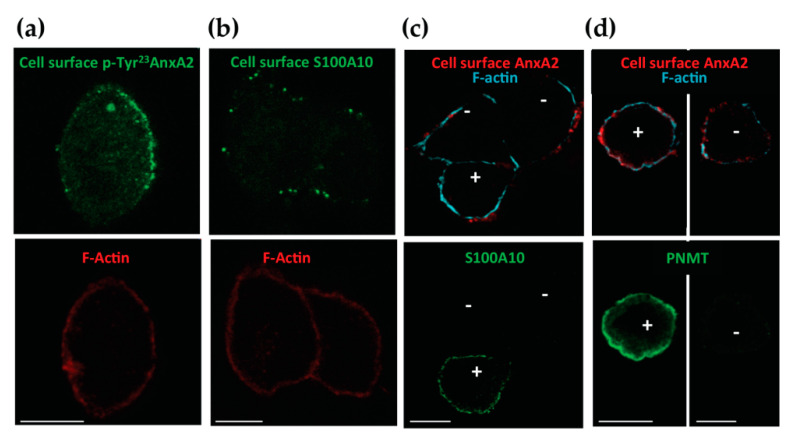
Tyr^23^-phosphorylated AnxA2 and S100A10 are associated with the surface of stimulated chromaffin cells. (**a**) The p-Tyr^23^AnxA2 labeling at the surface of stimulated chromaffin cells. Anti-p-Tyr^23^AnxA2 antibodies were revealed with Alexa Fluor^®^488-conjugated anti-mouse antibodies and F-actin with TRITC-phalloidin to visualize the cell shape. (**b**) S100A10 labeling at the surface of stimulated chromaffin cells. Anti-S100A10 antibodies were revealed with Alexa Fluor^®^488-conjugated anti-mouse antibodies and F-actin with TRITC-phalloidin. (**c**) Confocal micrograph of the triple labeling of cell surface AnxA2, of intracellular S100A10 and of F-actin labeled with Atto-647 *N*-phalloidin in stimulated chromaffin cells. (**d**) Confocal micrographs of the triple labeling of cell surface AnxA2, of intracellular PNMT and of F-actin labeled with Atto-647 *N*-phalloidin in stimulated chromaffin cells. For (**a**–**d**), confocal images were recorded in the same optical section by a dual exposure procedure. Scale bar: 10 µM.

In chromaffin cells, we have previously showed that AnxA2 can be found in monomeric and tetrameric forms, associated with two S100A10 molecules [19]. We examined whether cell surface AnxA2 was phosphorylated on Tyr^23^ and whether S100A10 was present on the cell surface upon cell stimulation. Live chromaffin cells were stimulated with nicotine in the presence of anti-pTyr^23^ AnxA2 or anti-S100A10 antibodies. Both antibodies labeled the surface of stimulated cells, suggesting that cell surface AnxA2 is Tyr^23^ phosphorylated and associated with S100A10. Thus, AnxA2 tetramer could be present on the external face of the plasma membrane (Figure 2a,b). Bovine chromaffin cells are constituted of two populations: the adrenergic cells secreting adrenaline and noradrenergic cells secreting noradrenaline [24], and we have previously showed that S100A10 is selectively expressed in adrenergic cells [14]. Thus, we performed a staining experiment for cell surface AnxA2 together with S100A10 or phenylethanolamine *N*-methyltransferase (PNMT), selectively expressed in adrenergic cells [24]. Figure 2c shows a S100A10-positive cell close to two S100A10-negative cells and all cells display a similar staining of cell surface AnxA2. Accordingly, cell surface AnxA2 labeling was observed on PNMT-positive and -negative cells (Figure 2d). Altogether, these data suggest that AnxA2 translocated through the plasma membrane of stimulated chromaffin cells even in the absence of S100A10.

### 3.3. The Egress of AnxA2 is Linked to Exocytosis

To probe the idea that AnxA2 egress is related to exocytosis, we labeled in parallel cell surface AnxA2 and the exocytotic sites using anti-DBH antibodies [14]. In live cells stimulated for exocytosis, the granule-associated DBH becomes transiently accessible to the antibody only at sites of exocytosis, leading to the appearance of fluorescent patches at the cell surface [14].

**Figure 3:**
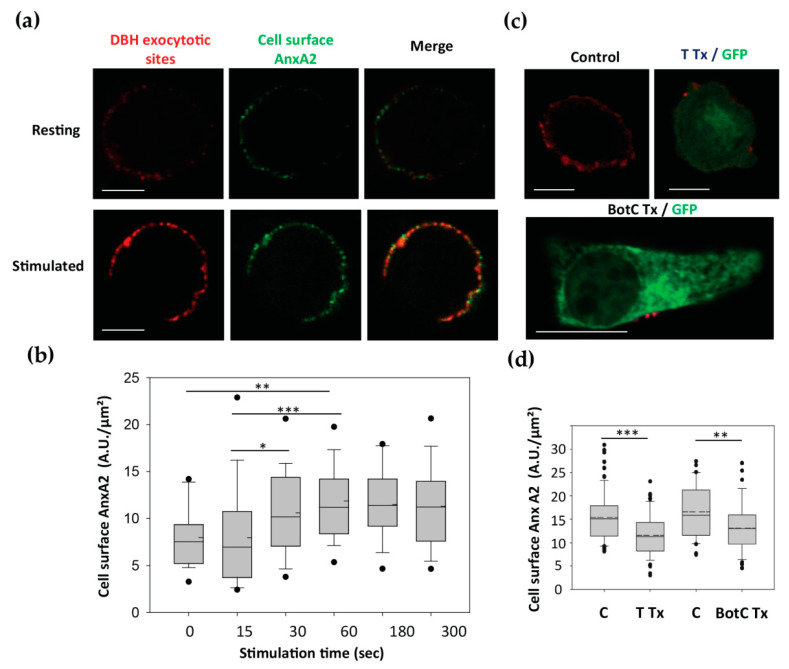
AnxA2 egress was correlated with exocytosis. (**a**) Dual labeling of cell surface AnxA2 and exocytotic sites. Cells were stimulated with nicotine 20 µM in the presence of anti-DBH and anti-AnxA2 antibodies. Cells were then fixed and incubated with secondary antibodies coupled to Alexa Fluor^®^561 and Alexa Fluor^®^488, respectively. Confocal images were recorded in the same optical section and with the same parameters of lasers and photomultipliers. Scale bar: 10 µM. (**b**) Time course of AnxA2 egress after cell stimulation. Chromaffin cells were stimulated with 20 µM nicotine during different times in the presence of AnxA2 antibodies. The cell surface AnxA2 labeling is expressed in arbitrary units. Statistical significance for medians was determined using a Mann-Whitney test. Dotted lines indicate the mean and asterisks statistical significance (*** = *p* < 0.001, ** = *p* < 0.01, * = *p* < 0.05). Two experiments were done on independent cell cultures and pooled. Number of cells analyzed were 19 (0 s), 25 (15 s), 29 (30 s), 23 (60 s), 21 (180 s). (**c**) Effect of tetanus and botulinum C toxins on the AnxA2 egress in chromaffin cells. Electroporated cells expressing TTx/GFP, BotC Tx/GFP or no toxin (Control, C) were stimulated with high K^+^ solution in the presence of anti-AnxA2 antibodies, fixed and then incubated with secondary antibodies coupled to Alexa Fluor^®^561. Confocal images were recorded in the same optical section. Scale bar: 10 µM. (**d**) Semi-quantitative analysis of cell surface AnxA2 labeling in stimulated cells is expressed in arbitrary units (Control *n* = 79 cells, TTx/GFP *n* = 69 cells, Control *n* = 39 cells, BotC Tx/GFP *n* = 39 cells). Statistical significance for medians was determined using a Mann-Whitney test. Dotted lines indicate the mean and asterisks statistical significance (*** = *p* < 0.001, ** = *p* < 0.01). Three experiments were done on independent cell cultures and pooled.

As illustrated in Figure 3a, resting chromaffin cells exhibited only a few DBH patches, confirming the low levels of baseline exocytotic activity in the absence of secretagogue, and displayed only a weak cell surface AnxA2 staining. Stimulation for 5 min with nicotine triggered the appearance of a patchy pattern of DBH surface staining and concomitantly increased cell surface AnxA2 labeling (Figure 3a). We also observed the co-localization between DBH and AnxA2 at the cell surface (Figure 3a, merge). Semi-quantitative analysis indicated that 65.5 ± 4.12% (±SEM, *n* = 27) of the cell surface AnxA2 labeling colocalized with DBH labeling. This indicated that the AnxA2 egress primarily takes place in the vicinity of the exocytotic sites. Next, we examined the time course of AnxA2 appearance on the cell surface of nicotine-stimulated chromaffin cells using a semi-quantitative analysis (Figure 3b). Egress of AnxA2 was observed after 30 s of stimulation and it peaked after 60 s. Thus, the kinetics of AnxA2 egress correlated well with the rapid kinetics of AnxA2 Tyr^23^ phosphorylation during exocytosis, but preceded the maximal exocytotic response usually observed after 180 s of stimulation in our experimental conditions [6]. To confirm the link between the AnxA2 egress and exocytosis, we examined whether the formation of the Soluble NSF Attachment Proteins REceptor (SNARE) complexes was necessary for the externalization of AnxA2. A bicistronic plasmid encoding for tetanus toxin (TTx) and GFP was expressed in chromaffin cells. TTx is known to specifically cleave VAMP2, a protein required for SNARE complex formation and exocytosis [25]. As illustrated in Figure 3c, cell surface AnxA2 labeling was reduced in stimulated cells expressing TTx as compared to electroporated control cells not expressing TTx. Semi-quantitative analysis confirmed that expression of TTx reduced AnxA2 egress in stimulated cells (Figure 3d). Thus, VAMP2 cleavage and the consequent inhibition of the SNARE complex formation reduced AnxA2 externalization and its appearance on the cell surface. Similar results were obtained in cells expressing botulinum toxin C, which cleaves SNAP-25 and syntaxin-1 (Figure 3c,d). These findings indicate that the egress of AnxA2 seems to depend on the formation of SNARE complexes and therefore on the completion of exocytosis in chromaffin cells.

### 3.4. Cell Surface Membrane-Associated t-PA is Present Close to Exocytotic Sites

**Figure 4:**
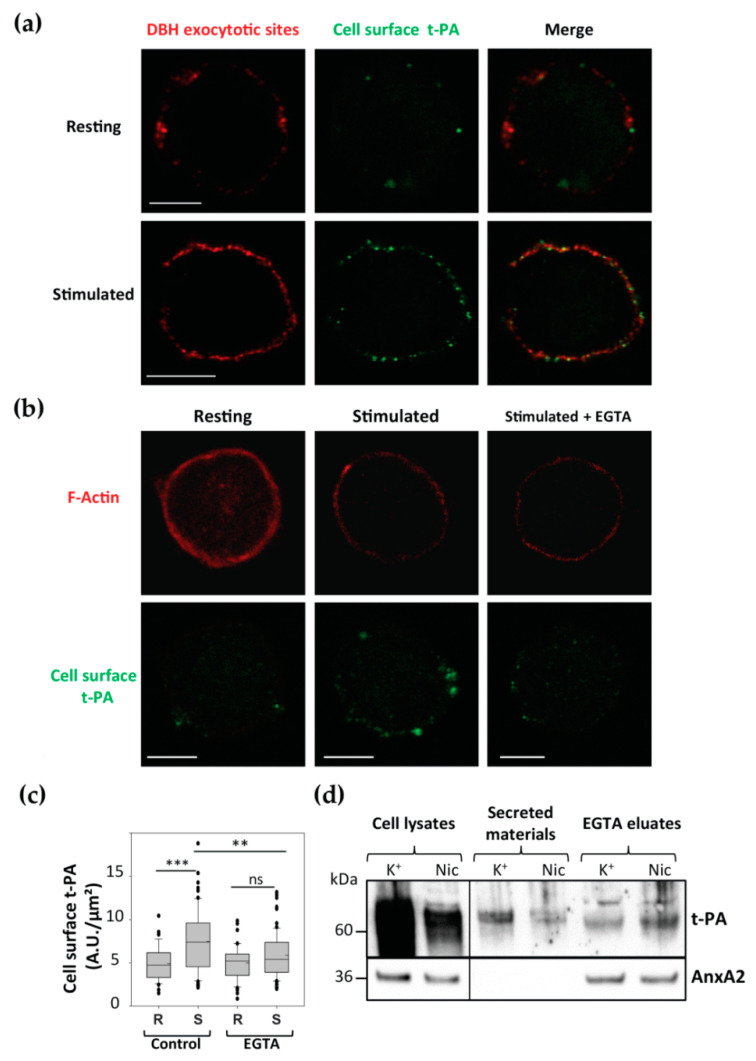
Tissue plasminogen activator (t-PA) is present on the outer leaflet of the plasma membrane of stimulated chromaffin cells. (**a**) Dual labeling of cell surface t-PA and exocytotic sites. Cells were stimulated with nicotine 20 µM in the presence of anti-t-PA and anti-DBH antibodies. Cells were then fixed and incubated with secondary antibodies coupled to Alexa Fluor^®^488 and Alexa Fluor^®^551, respectively. Confocal images were recorded in the same optical section and with the same parameters of lasers and photomultipliers. Scale bar: 10 µM. (**b**) The t-PA labeling on the surface of chromaffin cells in the resting condition (R), stimulated with 20 µM nicotine without (S) or after EGTA wash (S + EGTA). Anti-t-PA antibodies were revealed with Alexa Fluor^®^488-conjugated anti-rabbit antibodies and F-actin with TRITC-phalloidin to visualize the cell shape. Confocal images were recorded in the same optical section by a dual exposure procedure. Scale bar: 10 µM. (**c**) Semi-quantitative analysis of t-PA labeling on the cell surface of chromaffin cells in the resting condition (R), stimulated with 20 µM nicotine (S) without (Control) or after EGTA wash (S + EGTA). The t-PA labeling is expressed in arbitrary units. Statistical significance for medians was determined using a Mann-Whitney test. Dotted lines indicate the mean and asterisks statistical significance (*** = *p* < 0.001, ** = *p* < 0.01). Three experiments were done on independent cell cultures and pooled (*n* = 36 and 65 control cells, 40 and 75 EGTA-treated cells for resting and stimulated conditions, respectively). (**d**) Lysate, secreted material and EGTA eluate from chromaffin cells in the resting condition (R) or stimulated with nicotine 20 µM (S) were analyzed by western blot and revealed with anti-t-PA and anti-AnxA2 antibodies. Data correspond to a typical experiment representative of three independent experiments.

Cell surface AnxA2 has been described as a co-receptor of t-PA [26]. Cell surface AnxA2 tetramer was shown to capture circulating plasminogen and t-PA to promote plasmin generation that participates in fibrinolysis [26]. Moreover, previous studies reported the presence of t-PA in secretory granules and its release upon chromaffin cell stimulation [27], and t-PA was found to bind to the cell surface by interacting with an unknown receptor [28]. We confirmed the presence of t-PA at the surface of chromaffin cells (Figure 4). Double labeling of living cells stimulated with nicotine in the presence of anti-t-PA and anti-DBH antibodies revealed the appearance of a patchy pattern of DBH surface staining and a concomitant increase in t-PA labeling (Figure 4a). We also observed the co-localization between t-PA and DBH at the cell surface (Figure 3a, merge). Semi-quantitative analysis indicated that 68 ± 2.7% (±SEM, *n* = 33) of the cell surface t-PA labeling colocalized with DBH labeling.

We further characterized the binding of t-PA at the cell surface of stimulated chromaffin cells using a similar approach to that used to visualize cell surface AnxA2. Live cells were maintained at rest or stimulated with nicotine in the presence of anti-t-PA antibodies prior to fixation. In resting cells, t-PA labeling was barely visible, whereas in stimulated cells, a clear increase in cell surface labeling was observed (Figure 4b). Washing the nicotine-stimulated cells with calcium-free EGTA containing Locke’s solution prior to incubation with the anti-t-PA antibodies resulted in a clear reduction in t-PA labeling (Figure 4b). Semi-quantitative analysis (Figure 4c) confirmed that cell stimulation increased by approximately 2.5-fold the t-PA staining on the surface of chromaffin cells and validated the calcium sensitivity of t-PA binding to the plasma membrane. Figure 4d illustrates a western blot analysis of the fractions collected from cells stimulated with nicotine or high K^+^ solution and subsequently washed for 5 min with EGTA solution. In the cell lysates and secreted material, t-PA was found, in line with the presence of t-PA in secretory granules. It was also detected in the EGTA eluates, indicating that part of the t-PA released by exocytosis remained bound to the cell surface in a calcium-dependent manner. CgA, a major component stored in chromaffin granules and released by exocytosis, also bound to the cell surface following secretion (Figure 1c).

### 3.5. AnxA2 and t-PA are Side by Side at Cell Surface of Stimulated Chromaffin Cells

**Figure 5:**
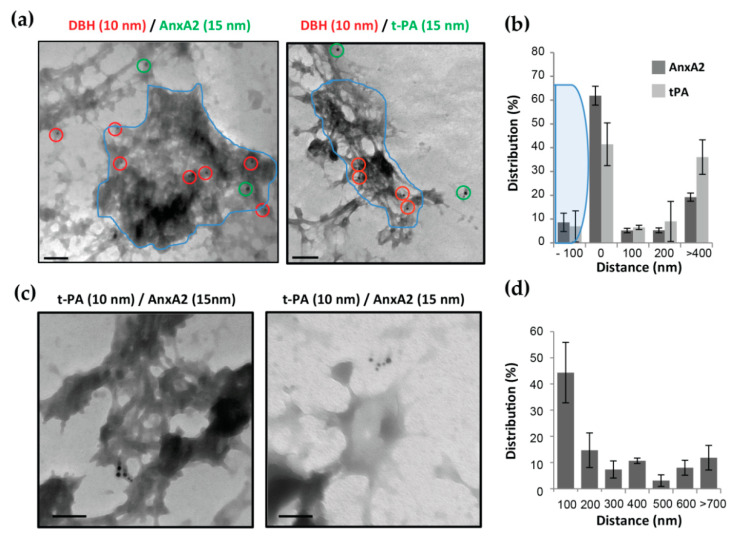
Membrane topography of AnxA2, t-PA and exocytotic sites after immunogold labeling of the outer face of the plasma membrane sheets prepared from stimulated chromaffin cells. (**a**) Plasma membrane sheets were prepared from bovine chromaffin cells stimulated by nicotine for 5 min. To label DBH, AnxA2 and t-PA exposed at the surface of cells undergoing exocytosis, anti-DBH, anti-AnxA2 and anti-t-PA antibodies were added during stimulation. Membrane sheets were labeled with anti-mouse antibodies coupled to 10 nm gold particles to detect DBH antibodies revealing exocytotic sites (red circle) and rabbit antibodies coupled to 15 nm gold particles to label AnxA2 or t-PA (green circle). (**b**) The histogram represents the relative distribution of 15 nm gold particles as a function of their distance from the granule membrane once inserted in the plasma membrane (blue line). The distance was measured and the number of particles was counted manually with Photoshop. Three experiments were done on independent cell cultures (**c**). Double staining experiment for t-PA (10 nm gold particles) and AnxA2 (15 nm gold particles) were performed with the same protocol. Scale bar: 100 nm. (**d**) The histogram represents the relative distribution of 10 nm gold particles (t-PA) as a function of their distance from 15 nm gold particles (AnxA2). The distance was measured and the number of particles was counted manually with Photoshop. Two experiments were done on two independent cell cultures.

To further explore the spatial relationship between t-PA, AnxA2 and the sites of exocytosis, experiments were designed to analyze at the ultrastructural level the outer face of the chromaffin cell plasma membrane. The localization of cell surface AnxA2 or t-PA and granule membranes transiently inserted into the plasma membrane after exocytosis was examined on plasma membrane sheets from chromaffin cells stimulated with 20 µM nicotine in the presence of anti-DBH antibodies to reveal exocytotic granule membranes [29] and anti-AnxA2 or anti-t-PA antibodies (Figure 5). Cells were fixed and labeled with anti-mouse antibodies revealed with 10 nm gold particles and anti-rabbit antibodies revealed with 15 nm gold particles to label DBH/anti-DBH complexes and AnxA2/anti-AnxA2 or t-PA/anti-t-PA, respectively. Ultrastructural images obtained by transmission electron microscopy revealed the appearance of gold-labeled DBH clusters on the cell surface (Figure 5a), corresponding to the insertion of the granular membrane in the plasma membrane of nicotine-stimulated cells [29]. Cell surface AnxA2, as well as t-PA (Figure 5a,b), tended to localize at the periphery of the DBH-labeled areas. Double-labeling experiments with anti-t-PA and anti-AnxA2 antibodies using a combination of 10 and 15 nm gold particles indicated AnxA2 and t-PA formed mixed clusters at the cell surface (Figure 5c,d). Both types of beads were found in close proximity, suggesting a possible interaction of t-PA with AnxA2 at the surface of stimulated chromaffin cells, in line with the idea that AnxA2 could be a t-PA receptor at the surface of chromaffin cells.

## 4. Discussion

During neuroendocrine secretion, AnxA2 participates together with the actin cytoskeleton in the formation of lipid micro-domains required for the docking and fusion of secretory granules with the plasma membrane [5,13,19]. We have recently observed that AnxA2 needs to be phosphorylated on Tyr^23^ to form these lipid platforms supporting exocytosis [6]. Since Tyr^23^ phosphorylation of AnxA2 has been reported to induce its translocation from the inner to the outer side of the plasma membrane [7], we examined here whether AnxA2 might be externalized in neurosecretory chromaffin cells. In agreement with these findings, the present report favors a model in which AnxA2 is not conventionally secreted in the extracellular medium, but rather translocates through the plasma membrane and remains attached to the surface of stimulated chromaffin cells. Here, p-Tyr^23^-AnxA2 was found associated with the outer face of the plasma membrane in a calcium-dependent interaction. Hence, our observations are in agreement with previous results obtained in endothelial cells [7] and keratinocytes [30], but also in neurosecretory GABAergic neurons [9]. AnxA2 egress is closely linked to the process of exocytosis since the externalization of AnxA2 required the formation of the SNARE complexes and occurred near the sites of secretory granule fusion. In addition, at the cell surface, AnxA2 was found to co-localize with t-PA in a calcium-dependent manner. We propose that cell surface AnxA2 could function as a receptor for secreted t-PA.

### 4.1. By Which Mechanism is AnxA2 Translocated to the Cell Surface in Chromaffin Cells?

Despite the description of many extracellular functions for AnxA2, the mechanism underlying its translocation across the plasma membrane to the cell surface remains unclear. There are a number of soluble proteins lacking signal peptides which are secreted in the extracellular medium through a process called “unconventional secretion” [31]. Two major pathways for this secretion involve either the direct translocation across the plasma membrane or the secretion via extracellular vesicles. For instance, in NIH 3T3 fibroblasts, the cell surface appearance of AnxA2 has been linked to the fusion of multi-vesicular bodies with the plasma membrane, whereas in intestinal epithelial cells, it depends on the fusion of secretory vesicles with the plasma membrane, and in stimulated macrophages or ultraviolet-irradiated keratinocytes it requires caspase-1 activation [32]. In chromaffin cells, the implication of secretory granules and extracellular vesicles is unlikely since AnxA2 was neither found in the soluble secreted material nor associated with the extracellular vesicles released after cell stimulation, but detected on the outer face of the plasma membrane where it bound in a calcium-dependent manner. Furthermore, we did not find AnxA2 in a 100,000 g pellet of the secreted material, suggesting that AnxA2 is not associated with the extracellular vesicles released after cell stimulation. Consequently, the most likely mechanism for AnxA2 membrane translocation involves direct insertion into the lipid bilayer, allowing the passage of AnxA2 through the plasma membrane.

The insertion of annexins into model membranes has been demonstrated in vitro for several members of this large family of proteins (AnxA1, A2, A4, A5 and A6) [33]. Within cells, AnxA2 was also shown to translocate across membranes [34]. AnxA2 membrane translocation requires calcium-dependent binding to negatively charged phospholipids such as phosphatidylserine (PS) and a lipid flipping activity. Accordingly, the phospholipid scramblase TMEM16F was reported to contribute to AnxA2 membrane crossing [35]. A similar mechanism has been described for the unconventional secretion of fibroblast growth factors (FGFs) [36], which also requires calcium and is linked to PS egress [37]. It is tempting to draw parallels with AnxA2 in chromaffin cells, since secretagogue-evoked stimulation of chromaffin cells triggers the appearance of PS at the cell surface, presumably at the periphery of the granule fusion sites [29,38]. This PS egress depends on the lipid scramblase PLSCR-1 [39]. The contribution of PLSCR-1 in AnxA2 egress needs now to be tested in chromaffin cells. Finally, an alternative mechanism has been proposed in enterocytes, which involves the extrusion of AnxA2 during hemifusion [40].

### 4.2. What Might be the Role of Cell Surface AnxA2?

The most well-documented role of cell surface AnxA2 is as co-receptor of t-PA and plasminogen [26]. Plasminogen and t-PA were previously described on the surface of chromaffin cells but their receptors remained unidentified [41]. Of note, t-PA was also detected in the secretory granules of a subpopulation of chromaffin cells [42]. In the present report, t-PA and AnxA2 were both found on the surface of chromaffin cells. Both t-PA and cell surface AnxA2 were eluted by EGTA, indicating that both proteins bind to the membrane in a calcium-dependent manner. Although the direct interaction of t-PA with chromaffin cell surface AnxA2 remains to be demonstrated, AnxA2 is able to interact with t-PA and its primary substrate, plasminogen [43]. Thus, by recruiting released t-PA and circulating plasminogen, cell surface AnxA2 might well serve as an extracellular proteolytic center that locally generates plasmin. Plasmin is known to cleave released CgA into a variety of biologically active peptides, some of which may significantly inhibit the nicotinic stimulation of catecholamine release from PC12 cells and primary bovine adrenal chromaffin cells [28]. For instance, the fragment CgA_360-373_ is selectively generated by plasmin and the corresponding synthetic peptide markedly inhibited nicotine-induced catecholamine release [44]. Plasmin generated at the cell surface could also activate signals, leading to protein kinase C-mediated phosphorylation of intracellular AnxA2, thereby dissociating the AnxA2 complex and preventing further catecholamine release [32]. In neuronal tissues, plasminogen activators and cell surface AnxA2 have both been detected at high levels and implicated in processes like neuronal plasticity and synaptic remodeling within the hippocampus and cerebellum during memory [10].

Additionally, AnxA2, via its ability to sequester and laterally organize PS [45], could promote the formation or stabilization of PS-rich domains in the external leaflet of the plasma membrane. These AnxA2-mediated clusters of PS at the cell surface may represent a concentrated signal for endocytosis as has been shown for AnxA5 and phagocytosis [46]. Finally, we cannot exclude that the AnxA2 tetramer could potentially interact with neighboring phospholipid membranes and therefore serve as a bridge between two adjacent cells [47].

To summarize, we propose that, upon cell stimulation, AnxA2 is recruited to the plasma membrane to form a tetramer with S100A10. Once phosphorylated at the plasma membrane, AnxA2 phosphorylated on Tyr^23^ stabilizes lipid micro-domains required to recruit/organize the priming/docking machinery for exocytosis. Although AnxA2 Tyr^23^ dephosphorylation is required to promote the formation of actin bundles that strongly anchor secretory granules to the exocytotic sites, a fraction of Tyr^23^-phosphorylated AnxA2 appears to cross the plasma membrane near exocytotic sites. At the cell surface, AnxA2 as a co-receptor of t-PA could thus participate in various autocrine and/or paracrine activities.
